# A role for Mitochondrial Rho GTPase 1 (MIRO1) in motility and membrane dynamics of peroxisomes

**DOI:** 10.1111/tra.12549

**Published:** 2018-02-20

**Authors:** Inês G. Castro, David M. Richards, Jeremy Metz, Joseph L. Costello, Josiah B. Passmore, Tina A. Schrader, Ana Gouveia, Daniela Ribeiro, Michael Schrader

**Affiliations:** ^1^ Biosciences University of Exeter Exeter UK; ^2^ LSI University of Exeter Exeter UK; ^3^ Institute of Biomedicine University of Aveiro Aveiro Portugal

**Keywords:** mathematical modelling, membrane protrusion, microtubule, MIRO1, organelle motility, peroxisome, proliferation

## Abstract

Peroxisomes are dynamic organelles which fulfil essential roles in lipid and ROS metabolism. Peroxisome movement and positioning allows interaction with other organelles and is crucial for their cellular function. In mammalian cells, such movement is microtubule‐dependent and mediated by kinesin and dynein motors. The mechanisms of motor recruitment to peroxisomes are largely unknown, as well as the role this plays in peroxisome membrane dynamics and proliferation. Here, using a combination of microscopy, live‐cell imaging analysis and mathematical modelling, we identify a role for Mitochondrial Rho GTPase 1 (MIRO1) as an adaptor for microtubule‐dependent peroxisome motility in mammalian cells. We show that MIRO1 is targeted to peroxisomes and alters their distribution and motility. Using a peroxisome‐targeted MIRO1 fusion protein, we demonstrate that MIRO1‐mediated pulling forces contribute to peroxisome membrane elongation and proliferation in cellular models of peroxisome disease. Our findings reveal a molecular mechanism for establishing peroxisome‐motor protein associations in mammalian cells and provide new insights into peroxisome membrane dynamics in health and disease.

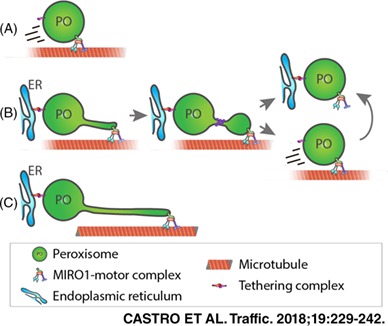

**Abbreviations**
TA: tail‐anchored
TMD: transmembrane domain
WT: wild type
ROS: reactive oxygen species
ER: endoplasmic reticulum

## 1 INTRODUCTION

Peroxisomes are dynamic, multifunctional organelles that vary in size, number and shape depending on cell type, environmental stimuli and metabolic demand,^1^ but the underlying molecular mechanisms which govern this versatility are not fully understood. Similar to mitochondria, peroxisomes are oxidative organelles that fulfil important functions in lipid metabolism and ROS homeostasis rendering them essential for human health and development.^2^, ^3^ Peroxisomes metabolically cooperate and physically interact with a variety of subcellular organelles including the ER, mitochondria, lipid droplets and other peroxisomes.^4^, ^5^, ^6^ These functions require peroxisome positioning and movement within eukaryotic cells.

Whereas in yeast and plant cells peroxisome motility depends on actin filaments and myosin motors,^7^, ^8^ in mammalian cells peroxisomes move bidirectionally via microtubules, using both kinesin and dynein motors.^9^, ^10^, ^11^, ^12^ The shape and number of peroxisomes is controlled by PEX11β, a peroxisomal membrane protein, which induces elongation and remodelling of the peroxisomal membrane and acts as a GTPase activating protein on the large fission GTPase DNM1L.^13^, ^14^, ^15^ Loss of PEX11β was recently linked to spindle misorientation and peroxisome mislocalisation in mitosis causing imbalances in epidermal differentiation.^16^ These findings underline the importance of peroxisome multiplication, distribution and inheritance for cell fate decisions.

Although key factors required for peroxisome dynamics and multiplication have been identified, it is currently unclear to what extent cytoskeletal tracks, docking factors and pulling forces mediated by associated motor proteins contribute to these processes, in particular in mammals.^17^ In baker’s yeast, peroxisome distribution and inheritance depends on actin, the myosin motor Myo2 and specific adaptor proteins, Inp1 and Inp2, at the peroxisomal membrane.^7^ Furthermore, the peroxins Pex3 and Pex19 have been found to interact with myosin motors.^18^, ^19^ In contrast, little is known about the recruitment of microtubule motors to peroxisomes in mammalian cells.^20^

Here, we identify the Ras GTPase MIRO1 as a potential adaptor for microtubule‐based peroxisome motility in mammalian cells. MIRO proteins were initially identified on the outer mitochondrial membrane^21^ where they, together with TRAK1/2, link the microtubule motors kinesin and dynein to mitochondria,^22^, ^23^, ^24^, ^25^ and play key roles in mitochondrial motility, homeostasis and inheritance.^26^, ^27^ Mammalian MIRO1 and MIRO2 share 60% similarity and an analogous structure containing 2 GTPase and 2 EF‐hand calcium binding domains.^21^, ^28^ Studies on mammalian MIRO proteins have focused mainly on MIRO1 due to its clear role in mitochondrial motility, particularly in neurons.^22^, ^25^ Loss of MIRO1‐directed mitochondrial movement and distribution result in neurological defects.^26^ MIRO1‐mediated mitochondrial positioning is also suggested to shape intracellular energy gradients required for cell migration.^29^ We show that MIRO1 localises to peroxisomes and mitochondria, and alters peroxisome distribution and motility. Furthermore, we demonstrate that an exclusively peroxisome‐targeted MIRO1 can mediate pulling forces which contribute to peroxisome membrane elongation and proliferation in a cell type‐dependent manner. To better understand the versatility of peroxisomes in mammalian cells, we build a first mathematical model of peroxisome dynamics. This model helps to explain the underlying principles of peroxisome morphologies induced by MIRO1‐mediated pulling forces and other factors which influence peroxisomal membrane dynamics.

## 2 RESULTS

### 2.1 MIRO1 is dually targeted to peroxisomes and mitochondria

**Figure 1:**
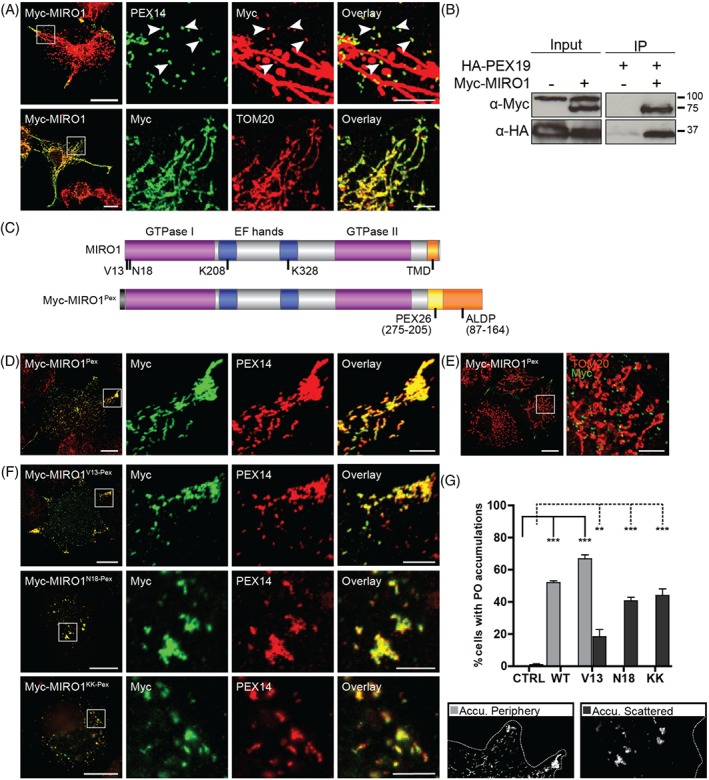
MIRO1 is targeted to peroxisomes and alters their distribution in COS‐7 cells. A, COS‐7 cells were transfected with Myc‐MIRO1, fixed and stained against Myc, and PEX14 or TOM20. B, Co‐immunoprecipitation from COS‐7 cells expressing HA‐PEX19 and Myc‐MIRO1, using α‐Myc‐conjugated agarose beads. HA‐PEX19 only co‐immunoprecipitated in the presence of Myc‐MIRO1. Higher band in α‐Myc Input is unspecific. Input—10% of total cell lysates, IP—immunoprecipitation. C, Schematic view of MIRO1 domains and mutation sites and the Myc‐MIRO1^PEX^ construct. D‐F, COS‐7 cells were transfected with Myc‐MIRO1^PEX^ constructs and stained against Myc and PEX14 or TOM20; D, Myc‐MIRO1^Pex^ was exclusively targeted to peroxisomes and induced redistribution to the cell periphery (E) without affecting mitochondrial morphology and distribution; F, mutated Myc‐MIRO1^Pex^ proteins were exclusively targeted to peroxisomes and induced the formation of peroxisomal accumulations in the cell periphery (V13) or scattered (V13, N18 and KK). G, Quantitative analysis of peroxisome distribution as shown in D‐F. Cells with peroxisomal accumulations in the periphery or scattered were counted. Values represent mean ± SEM of 3 independent experiments (100 replicates per experiment per condition; ** *P* < .01; *** *P* < .001; one‐way ANOVA with post hoc Tukey test vs control). Bars, 20 μm (overview), 5 μm (magnification)

Previous studies revealed a dual mitochondrial and peroxisomal localisation of several C‐tail‐anchored (TA) membrane proteins including FIS1, MFF and GDAP1, which function in peroxisomal and mitochondrial division.^30^, ^31^, ^32^, ^33^ In a recent study on the targeting of TA proteins to different organelles, we provided preliminary evidence for a dual peroxisomal and mitochondrial localisation of the Ras GTPases MIRO1 and MIRO2.^34^ MIRO1 was initially identified on the outer mitochondrial membrane,^21^ and forms a protein complex with TRAK1/2 that includes both kinesin and dynein motors, promoting mitochondrial movement through the microtubule cytoskeleton.^22^, ^23^, ^24^, ^25^ A dual mitochondrial and peroxisomal localisation of MIRO1 was confirmed by immunofluorescence after expression of Myc‐MIRO1 in COS‐7 cells (Figure 1A). Furthermore, we previously reported endogenous MIRO1 in highly purified peroxisomal and mitochondrial fractions,^34^ in agreement with proteomics data.^35^, ^36^

The targeting of all known TA proteins to peroxisomes requires the peroxisomal import receptor/chaperone PEX19.^34^ For MIRO1, PEX19 binding was shown by immunoprecipitation after co‐expression of Myc‐MIRO1 and HA‐PEX19 in COS‐7 cells (Figure 1B) suggesting a role for PEX19 in the targeting of MIRO1 to peroxisomes. Additionally, in a high‐throughput interaction study, MIRO1 was identified as a PEX19 interaction partner.^37^ These findings are also consistent with the known organelle targeting signals: MIRO1 possesses a transmembrane domain (TMD) with relatively low hydrophobicity (GRAVY, 1.3) and a moderate net charge in the tail region (1.9), which based on our previous work would be indicative of a TA protein that localises predominantly to mitochondria but has a potential for peroxisomal targeting.^34^ Overall, our findings support a dual localisation of MIRO1 at mitochondria and peroxisomes.

### 2.2 MIRO1 alters peroxisome distribution in COS‐7 cells

MIRO1 has been shown to play a key role in mitochondrial motility and distribution in mammalian cells.^26^ To determine if MIRO1 also plays a role in peroxisome positioning we expressed Myc‐tagged wild type (WT) and mutated versions in COS‐7 cells, and analysed their effect on peroxisome distribution (Figures 1A,C and S1). As previously described,^21^, ^38^ the expression of Myc‐MIRO1 resulted in abnormal mitochondrial morphologies (Figures 1A and S1). To avoid potential secondary effects due to dysfunctional mitochondria, we generated an exclusively peroxisomal set of MIRO1 proteins by altering the C‐terminal TMD using a previously described PEX26/ALDP construct (Figure 1C).^39^ Expression of the resulting Myc‐MIRO1^Pex^ fusion protein in COS‐7 cells revealed an exclusively peroxisomal localisation, with no effects on mitochondrial morphology and distribution (Figure 1D,E). Peroxisomes in COS‐7 cells usually distribute uniformly throughout the cytoplasm.^30^, ^40^ Interestingly, expression of Myc‐MIRO1^Pex^ or Myc‐MIRO1^V13‐Pex^, a constitutively active GTPase mutant, induced peroxisome redistribution and accumulation at the cell periphery (Figure 1D,F,G). On the other hand, expression of dominant negative Myc‐MIRO1^N18‐Pex^ and EF‐hand mutant Myc‐MIRO1^KK‐Pex^ resulted in peroxisome accumulations which were scattered throughout the cytoplasm (Figure 1F,G). Comparable results were obtained with the dually targeted MIRO1 versions (Figure S1B). Myc‐MIRO1^ΔTM^, a version lacking the TMD/tail sequence, localised to the cytoplasm and had no effect on peroxisome distribution, indicating that membrane anchorage is required for MIRO1 function (Figure S1). Furthermore, depolymerisation of microtubules with nocodazole in Myc‐MIRO1^Pex^ expressing cells abolished accumulation of peroxisomes in the cell periphery, suggesting that an intact microtubule cytoskeleton is required for peroxisome distribution via MIRO1 (Figure S2A). Our findings indicate that, similar to its role on mitochondria, MIRO1 can alter peroxisome distribution and positioning by affecting microtubule‐dependent peroxisome motility.

### 2.3 Peroxisomal MIRO1 increases movement of peroxisomes

**Figure 2:**
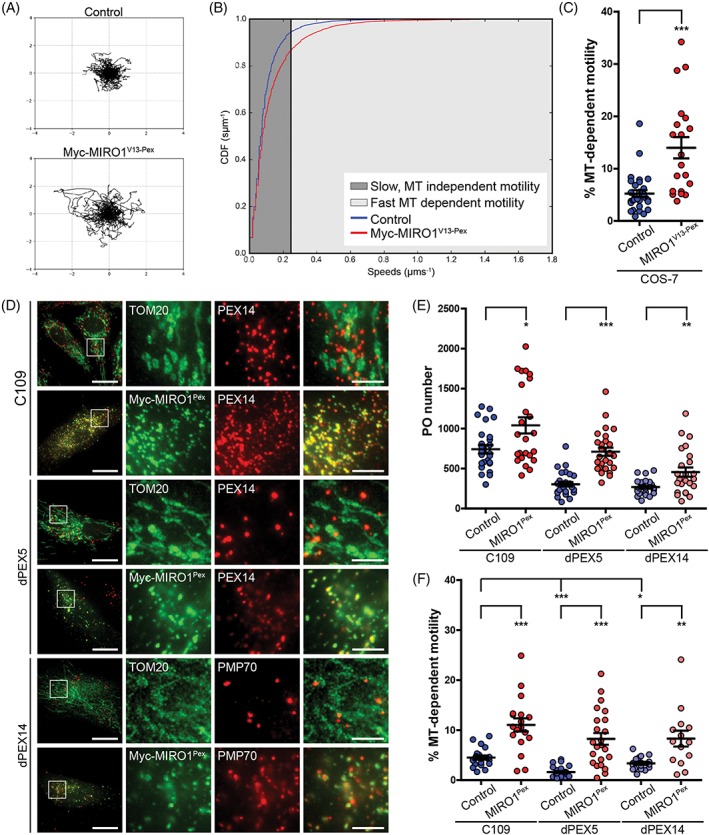
MIRO1 expression increases microtubule‐dependent peroxisome motility in mammalian cells and induces peroxisome proliferation in human skin fibroblasts. A‐C, Myc‐MIRO1^V13‐Pex^ expression increases peroxisome movement. COS‐7 cells were transfected with Myc‐Miro1^V13‐Pex^ and EGFP‐SKL. For each cell, 500 stacks of 5 planes were obtained over time, and peroxisomes detected and tracked using an automated algorithm. A, Trajectory plots. Hundred peroxisome trajectories were retrieved for each condition and the first 20 time‐frames plotted starting at a centre. B, CDF plot. Instantaneous trajectory speed profiles were estimated by calculating distance moved between each time point in the trajectory. These speeds were pooled and converted to an ECDF. By pooling speeds for all data sets for a given condition, a single ECDF was generated for each. A threshold of 0.24 μm/s was defined for microtubule‐dependent motility. C, Percentage of fast moving peroxisomes per cell in control and Myc‐MIRO1^V13‐Pex^ expressing cells. Values represent mean ± SEM of 20 to 30 cells from 3 independent experiments (*** *P* < .001; two‐tailed unpaired *t* test vs control cells). D, C109, dPEX5 and dPEX14 cells were transfected with Myc‐MIRO1^Pex^, fixed and stained against PEX14, TOM20, PMP70 and Myc. Expression of Myc‐MIRO1^Pex^ induces peroxisome proliferation. E‐F, C109, dPEX5 and dPEX14 cells were transfected with EGFP‐ACBD5^TMD‐T^ (peroxisomal membrane marker) alone, or co‐transfected with Myc‐MIRO1^Pex^. For each cell analysed, 250 stacks of 9 planes were obtained over time, and peroxisomes were detected and tracked using an automated algorithm. E, Quantitative analysis of peroxisome number (first stack of each tracked cell). In all cases, expression of Myc‐MIRO1^Pex^ significantly increased peroxisome number: C109‐741 ± 53 vs 1040 ± 101, dPEX5‐304 ± 27 vs 710 ± 51 and dPEX14‐268 ± 18 vs 457 ± 58. Values represent mean ± SEM of 24 to 29 cells from 3 independent experiments (* *P* < .05; ** *P* < .01; *** *P* < .001; one‐way ANOVA with post hoc Tukey test vs controls). F, Percentage of fast moving peroxisomes per cell in control and Myc‐MIRO1^Pex^ expressing fibroblasts. In all cases, peroxisome motility was significantly increased upon MIRO1 expression: C109‐4.51 ± 0.43 vs 11.05 ± 1.32, dPEX5‐1.61 ± 0.20 vs 8.25 ± 1.17, dPEX14‐3.36 ± 0.30 vs 8.30 ± 1.59. Values represent mean ± SEM of 14 to 26 cells in 3 independent experiments. Bars, 20 μm (overview), 5 μm (magnification)

To quantify the effect of MIRO1 expression on peroxisome motility, live‐cell imaging experiments were performed with COS‐7 cells expressing Myc‐MIRO1^V13‐Pex^ and the peroxisome marker EGFP‐SKL (Figure 2A‐C; Videos S1 and S2). To measure movement, peroxisomes were automatically detected and tracked using a customised in‐house algorithm.^41^ To visualise displacement, 100 trajectories were randomly sampled and plotted from a central point (Figure 2A). Expression of Myc‐MIRO1^V13‐Pex^ significantly increased peroxisome displacement. Figure 2B displays the empirical cumulative distribution function (ECDF) of the instantaneous peroxisome speeds for all peroxisomes analysed with each point of the curve corresponding to a single movement. For this analysis, all speed values above 0.24 μm/s were considered microtubule‐dependent movements as previously described.^42^ A significant increase in the number of fast moving peroxisomes can be observed in cells expressing Myc‐MIRO1^V13‐Pex^ (Figure 2B,C; Videos S1 and S2). Whereas in control cells 5.2% ± 0.7% of peroxisomes moved in a microtubule‐dependent manner, in cells expressing Myc‐MIRO1^V13‐Pex^ this number increased to 14.0% ± 2.0% (Figure 2C). Imaging of peroxisome accumulations at the cell periphery revealed that while the organelles appear to be confined to a relatively restricted area of the cell, peroxisomes regularly move within these accumulations, revealing dynamic interactions (Video S2). To examine the effect of a loss of MIRO1 function on peroxisome distribution and motility, we analysed MIRO1 KO MEFs (Figure S2B‐E). These cells have an altered mitochondrial distribution but peroxisome morphology and distribution appeared to be unaffected.^43^ In agreement with those findings, we did not detect any alterations in peroxisome distribution (Figure S2C) or motility (Figure S2D,E). These findings indicate that when targeted to peroxisomes in COS‐7 cells, active MIRO1, a known adaptor for the microtubule plus‐end motor kinesin, can redistribute peroxisomes to the cell periphery (where microtubule plus ends are located) in a microtubule‐dependent manner. However, MIRO1 may not be the only adaptor for microtubule‐dependent motor proteins at peroxisomes, as its loss is apparently not essential to maintain peroxisome distribution and motility. It is possible that MIRO2, which also localises to peroxisomes,^34^ can complement loss of MIRO1. Furthermore, peroxisomes may tether to or “hitch‐hike” other moving organelles to maintain their distribution. The latter process has been observed in filamentous fungi.^44^

### 2.4 MIRO1 induces peroxisome proliferation in human skin fibroblasts

The peroxisome‐targeted MIRO1 represents a new tool to manipulate peroxisome motility and to exert motor‐driven pulling forces at peroxisomes under control and disease conditions. Peroxisomes in fibroblasts from patients with peroxisomal disorders are often enlarged and reduced in number, and tend to cluster and detach from microtubules.^45^ We first expressed Myc‐MIRO1^Pex^ in human skin fibroblasts from a healthy control and examined its effect on the peroxisomal compartment (Figure 2D). Surprisingly, in these cells peroxisomes did not accumulate at the cell periphery but instead proliferated, presenting a significant increase in number (mean peroxisome number/cell: control 740 ± 50; Myc‐MIRO1^Pex^ 1040 ± 100, *n* = 24; Figure 2E). In addition, the percentage of motile peroxisomes that moved in a microtubule‐dependent manner was significantly increased (Figure 2F; Figure S2F; Videos S3 and S4). These findings indicate that MIRO1‐bound motor proteins can exert forces at peroxisomes, which result in peroxisome division, thus increasing peroxisome number. Separation by pulling forces is only possible when the peroxisome is tethered to another structure, as it would otherwise simply move in the direction of the pulling force (Figure 4B). This untethered motion is observed in COS‐7 cells, where MIRO1 expression accumulates peroxisomes in the cell periphery where microtubule‐plus ends are located (Figures 1 and 4B). We recently revealed that peroxisome‐ER membrane contacts are mediated by peroxisomal ACBD5 that interacts with ER‐resident VAPB to form a peroxisome‐ER tether.^46^ Loss of ACBD5 increased the movement of peroxisomes in human skin fibroblasts, indicating that peroxisome‐ER membrane contacts restrict peroxisome motility. In line with this, our analyses reveal that the percentage of fast moving peroxisomes in control fibroblasts is lower than that in control COS‐7 cells (4.5% ± 0.4% vs 5.2% ± 0.7%). We suggest that peroxisome‐ER tethering is cell‐type specific and that MIRO1/motor‐mediated pulling forces can induce peroxisome proliferation in fibroblasts, whereas in COS‐7 cells peroxisomes are dragged towards the cell periphery (Figure 4B). These findings indicate that a close interplay between tethering and motile forces modulates not only peroxisome distribution but also proliferation.

To analyse the impact of MIRO1 expression on peroxisomes in patient fibroblasts, we expressed Myc‐MIRO1^Pex^ in PEX5 and PEX14 deficient cells. PEX5 and PEX14 are proteins of the peroxisomal matrix protein import machinery, and loss of function leads to “empty” membrane structures (so called “ghosts”) that lack peroxisomal enzymes and are metabolically inactive. Peroxisomes in those cells are often enlarged and reduced in number (Figure 2D). Expression of Myc‐MIRO1^Pex^ in both PEX5 and PEX14 deficient cells induced peroxisome proliferation, but many peroxisomes remained enlarged (Figure 2D,E). MIRO1 expression also significantly increased peroxisome motility in patient cells (Figure 2F; Figure S2G‐H), most prominently for the smaller peroxisomes (Videos S5‐S8). In contrast to a recent report, we observed that peroxisomes in PEX14 deficient cells are motile.^47^ These findings show that MIRO1‐mediated pulling forces can at least partially induce the proliferation of metabolically inactive peroxisomes, indicating that membrane components are the most relevant factors for this process.

### 2.5 Peroxisome‐targeted MIRO1 promotes the formation of extended membrane protrusions in PEX5 deficient fibroblasts

**Figure 3:**
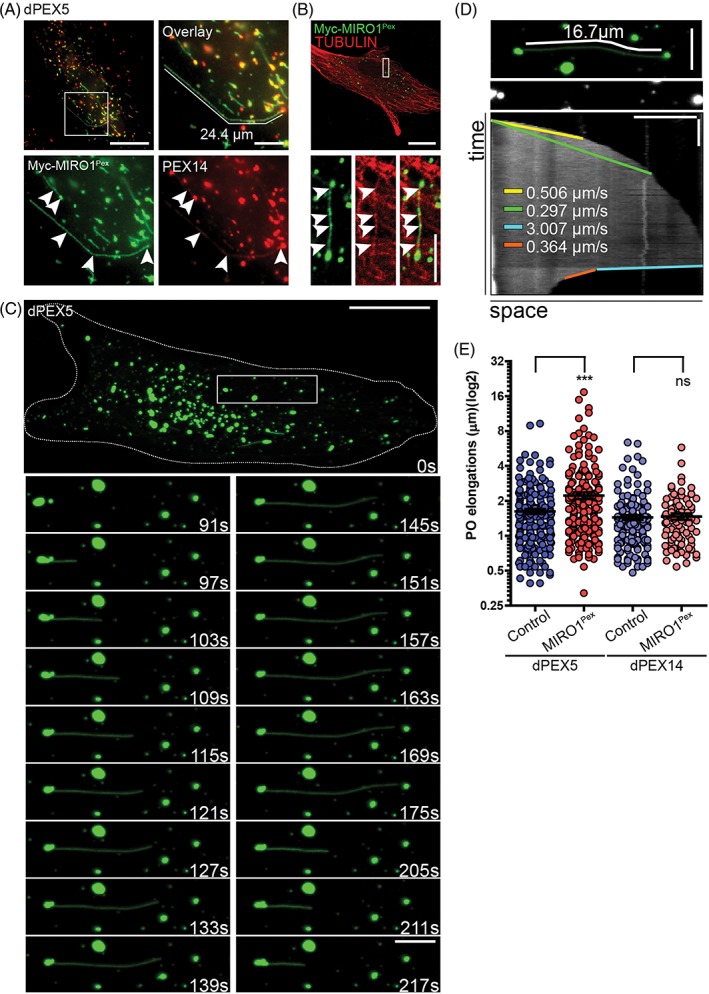
Expression of MIRO1 increases the length of peroxisome elongations in dPEX5 patient fibroblasts. A and B, dPEX5 patient fibroblasts were transfected with Myc‐MIRO1^Pex^, fixed and stained against (A) Myc and PEX14. The majority of observed elongations in fixed cells show an evenly distributed Myc‐MIRO1^Pex^ signal (arrowheads), likely originating from a large peroxisome as shown by the strong PEX14 staining at one of the extremities (arrowheads). B, dPEX5 cells were fixed and stained against PEX14 and TUBULIN. Elongated peroxisomal structures were usually found overlaying microtubules (arrowheads). C‐E, dPEX5 patient fibroblasts were transfected with EGFP‐ACBD5^TMD‐T^ (peroxisomal membrane marker) and Myc‐MIRO1^Pex^. C, Time lapse of peroxisome elongation forming and retracting in a dPEX5 cell expressing EGFP‐ACBD5^TMD‐T^ and Myc‐MIRO1^Pex^. D, Kymograph of peroxisome elongation observed in C. Bars, 20 seconds (vertical), 5 μm (horizontal). E, Quantitative analysis of peroxisome elongation length in dPEX5 and dPEX14 cells. Expression of Myc‐MIRO1^Pex^ significantly increased the length of peroxisome elongations in dPEX5 cells (1.62 ± 0.08 vs 2.21 ± 0.15), but not in dPEX14 cells (1.44 ± 0.07 vs 1.47 ± 0.09). Values represent mean ± SEM from 22 to 29 cells, 3 independent experiments (*** *P* < .001; two‐tailed unpaired *t* test vs controls). Bars, 20 μm (overview), 5 μm (magnification)

**Figure 4:**
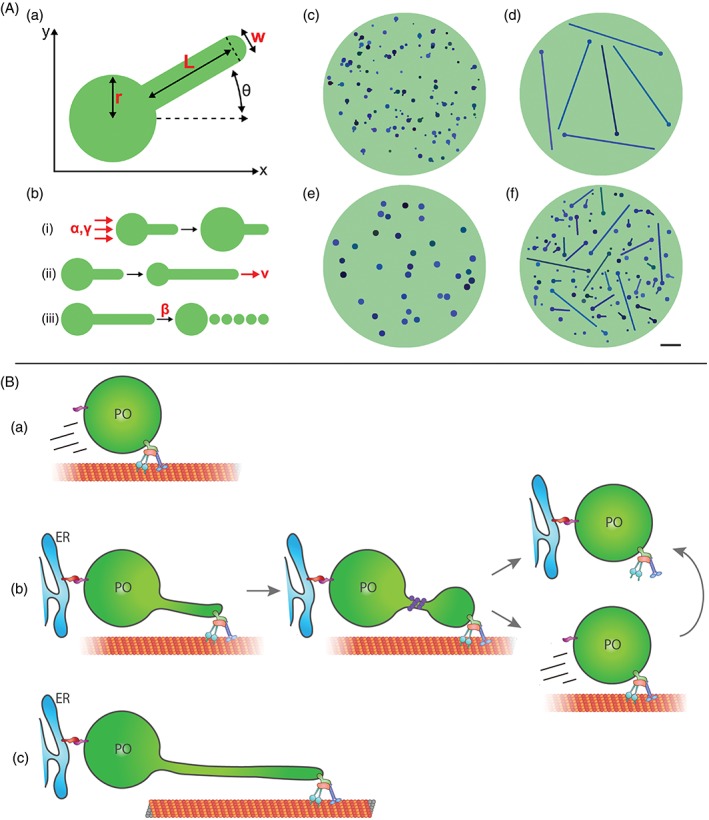
A, Mathematical model of peroxisomal growth and division. (a) Each peroxisome is represented as a spherical body of radius *r* and a cylindrical elongation of length *L* and diameter *w.* (b) The 3 processes implemented in the model: (1) membrane lipid flow into the body with rate *α* and lipid flow constant *γ*, (2) growth of the elongation at speed *v* and (3) division with rate per unit length *β.* (c) Snapshot from the model simulation of wild‐type cells (*α* = 75 nm^2^/s, *β* = 2 × 10^−5^/nm/s, *v* = 0.3 nm/s, *τ* = 40 hours, *γ* = 2.4 × 10^−7^/nm^2^). (d) Snapshot from the simulation of dMff cells (with reduced division rate β = 5 × 10^−11^/nm/s). (e) Snapshot from the simulation of dPex5 cells (β = 10^−9^/nm/s, v = 2 × 10^−4^ nm/s). (f) Snapshot from overexpression of MIRO1 in dPex5 cells (β = 10^−9^/nm/s, v = 3 nm/s, γ = 1.2 × 10^−7^/nm^2^). Bar, 1 μm. B, Schematic representation of the effects of MIRO1 on peroxisome dynamics and morphology. (a) Un‐tethered peroxisomes move via the microtubule cytoskeleton in a MIRO1 dependent manner; (b) peroxisomes tethered to the ER are pulled by MIRO1‐mediated motor forces and divide to form new peroxisomes; (c) defects in peroxisomal metabolism compromise MIRO1‐mediated peroxisome division and proliferation resulting in elongated membrane protrusions

Peroxisomes are highly dynamic organelles that can be found as spherical or elongated structures and also form membrane protrusions. These membrane alterations are suggested to contribute to peroxisome formation via division of elongated organelles, and to enable organelle crosstalk.^1^, ^48^ To what extent microtubule motors and pulling forces contribute to peroxisome membrane dynamics is unclear, as peroxisome elongation is unexpectedly promoted by microtubule‐depolymerising drugs, and peroxisome division can occur in the absence of microtubules.^9^, ^49^ In PEX5 deficient fibroblasts expressing Myc‐MIRO1^Pex^, we observed long membrane protrusions emanating from large, spherical peroxisomes and following linear tracks with sporadic bends (Figure 3A). These membrane protrusions co‐localised with microtubules, indicating their formation is promoted by MIRO1/motor generated pulling forces along microtubules (Figure 3B). Our observations also suggest the existence of as yet unidentified docking proteins which link peroxisomes to microtubules, and would facilitate the bending and directional changes we observe in the membrane protrusions. To analyse the dynamics of peroxisomal membrane protrusions, we performed time‐lapse analyses of PEX5 deficient cells expressing Myc‐MIRO1^Pex^ (Figure 3C). We revealed that protrusions originating from large peroxisomes grow at varying speeds, generally form straight lines in a single direction (Figure 3C‐E; Video S9) and occasionally appear to interact with other peroxisomes (Figure 3C). Transient peroxisome interactions, which may contribute to organelle crosstalk, have been previously reported.^42^ Interestingly, these protrusions can sometimes quickly retract, suggesting that the peroxisomal membrane has elastic properties that are largely unexplored (Figure 3C,D). A comparison of the surface area of a globular peroxisome from Zellweger fibroblasts (approximately 1 μm in diameter)^50^ with an elongated membrane protrusion (approximately 20 μm in length, 80 nm in diameter)^14^, ^46^ indicates a 16‐fold increase in the surface area of the protrusion. As it is unlikely that the globular peroxisome on its own can provide sufficient membrane lipids to generate such a protrusion, additional membrane lipids are likely provided by the ER. In support of this, we have recently revealed that peroxisome‐ER membrane contacts have an impact on peroxisomal membrane expansion.^46^ Whereas protrusions were not observed in control fibroblasts, they were formed in PEX5 and PEX14 deficient cells under control conditions, albeit more frequently in PEX5 deficient cells (Figure 3E). As peroxisomes are reduced in number in PEX5 and PEX14 deficient cells and are less motile than in controls, peroxisome protrusions may form to overcome those restrictions and to maintain organelle crosstalk. However, peroxisome metabolism is required to generate cellular lipids which are also necessary for peroxisome division and proliferation.^51^ As PEX5 and PEX14 deficient cells lack peroxisomal metabolic functions, their ability to divide and proliferate peroxisomes is compromised, which may explain the formation and frequency of membrane elongations (Figure 4B). Expression of Myc‐MIRO1^Pex^, however, significantly increased the frequency and length of protrusions in PEX5 deficient but not in PEX14 deficient cells (Figure 3E), suggesting that loss of PEX14 could interfere with the stability of membrane protrusions.

### 2.6 A mathematical model of peroxisome dynamics

To further understand the mechanisms involved in peroxisome dynamics, we developed a simple mathematical model that describes their growth and division. We used a stochastic, population‐based modelling approach that describes the morphology of a group of individual peroxisomes. Each peroxisome consists of a body of radius *r* with an optional elongation of length *L* and diameter *w* (Figure 4A(a)). The size of the body and elongation are controlled by 3 basic processes (Figure 4A(b)): (1) a membrane lipid flow rate to the body (eg, from the ER) (governed by rate *α* and lipid flow constant *γ*), (2) an elongation growth rate (governed by speed *v* and minimum radius *r*_min_) and (3) a division rate proportional to the elongation length (governed by rate *β* and minimum length *L*_min_). In addition, peroxisome turnover is controlled by the peroxisome mean lifetime *τ.* This leads to a model that is applicable to a range of experimental conditions (see Supporting information for full model details). Using WT parameters, we obtained a phenotype that reflects the heterogeneous peroxisome population observed in mammalian cells in terms of number, average body size and average elongation length (Figure 4A(c)). The WT division rate *β* is sufficiently high, resulting in division of peroxisome elongations shortly after formation. When considering a block in peroxisome division by setting the division rate *β* to almost zero, the model exhibits reduced numbers of peroxisomes all of which contain long elongations (Figure 4A(d)). Such a scenario is observed in patient fibroblasts lacking MFF, the membrane adaptor for the fission GTPase Drp1, where we would expect division rates to be significantly reduced.^52^, ^53^ The fact that changing only one parameter can capture this dramatic change in phenotype gives confidence that the model is able to correctly describe the basic processes involved in peroxisomal growth and division.

Next, we examined overexpression of MIRO1 in WT cells. For fibroblasts, we modelled this as a large increase (by a factor of 10) in the elongation growth rate *v* accompanied by an increase in lipid flow (modelled by halving the lipid flow constant *γ*). This leads to an increase in peroxisome number without a noticeable change in morphology, which is again explained by the fact that the WT division rate *β* causes almost all elongations to divide soon after formation, so that increased elongation growth rate and lipid flow can only result in proliferation (Figures 2D and 4B(b)). Conversely, in COS‐7 cells, MIRO1 overexpression results in peroxisomes moving to the cell periphery (Figures 1 and 4B(a)). We model this as an increase in *v* with no corresponding increase in lipid flow (eg, due to reduced peroxisome‐ER contact). Since lipid flow cannot keep up with the increased elongation speed, there is little impact on morphology or number, in agreement with our experimental observations.

**Figure 5:**
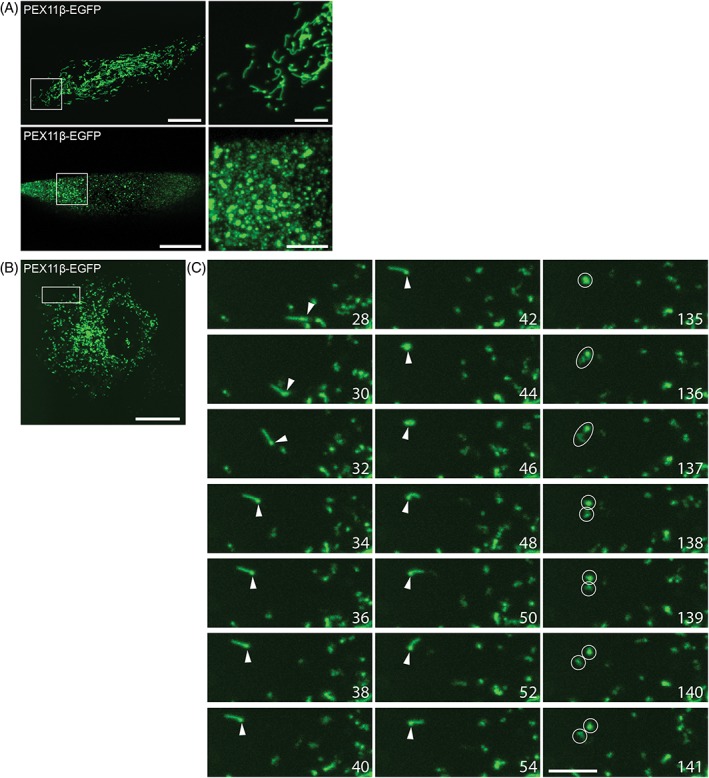
PEX11β promotes peroxisome membrane elongation and division. (A) PEX5 deficient patient fibroblasts or (B‐C) COS‐7 cells were transfected with PEX11β‐EGFP. A, PEX11β‐EGFP induces peroxisome proliferation, leading to the formation of elongated peroxisomes (top), followed by their fission into numerous small peroxisomes (bottom). C, Time lapse of peroxisome elongation (left) and division (right). Note the directed, long‐range movement of a peroxisome (arrow) with the linear protrusion leading (28‐40 seconds). The same peroxisome becomes static, whereas the membrane protrusion exhibits a more random, tentacle‐like movement (42‐54 seconds) before it divides (135‐141 seconds) (circles) (see also Video S10). For each cell analysed, 200 stacks of 9 planes were obtained over time. Time in seconds. Bars, 20 μm (overview), 5 μm (magnification)

The peroxisome phenotype in PEX5 deficient cells can be captured in the model by reducing both the division rate *β* and the elongation speed *v* (Figure 4A(e)), resulting in fewer and larger peroxisomes. This is in line with compromised peroxisome division and proliferation due to impaired peroxisomal lipid metabolism.^51^ Modelling overexpression of MIRO1 in PEX5 deficient cells (by also increasing *v* and decreasing *γ*) recapitulates the phenotype we found experimentally, where a substantial proportion of peroxisomes contain long elongations (Figure 4A(f),B(c)). Interestingly, this indicates that, despite a lack of peroxisomal metabolism, peroxisomal membranes retain their plasticity allowing lipid flow and membrane growth. Peroxisome proliferation in those cells is likely impaired due to reduced division rates (eg, due to altered peroxisomal membrane lipid composition). Whereas expression of MIRO1 cannot fully restore division, this can be achieved by PEX11β, a key factor in peroxisomal membrane remodelling and division^1^ (Figure 5A). Expression of PEX11β‐EGFP in PEX5 deficient cells promoted peroxisome elongation and subsequent division (Figure 5A) confirming earlier reports that PEX11β‐induced peroxisome proliferation is independent of peroxisomal metabolism.^45^, ^54^ As PEX11β also activates the fission GTPase Drp1,^15^ it likely increases the division rate *β* as well as the elongation speed *v* and lipid flow rate.

In mammalian cells, peroxisomes can elongate independently of microtubules, and peroxisome elongation is promoted by microtubule‐depolymerizing drugs.^9^, ^49^ This suggests that PEX11β and motor forces (eg, mediated by MIRO1) can act independently to promote peroxisome proliferation, but may cooperate under physiological conditions. This assumption is supported by live‐cell imaging of peroxisome dynamics in COS‐7 cells expressing PEX11β‐EGFP (Figure 5B,C).^14^ Similar to PEX5 deficient cells, PEX11β‐EGFP expression results in the formation of membrane protrusions emanating from globular peroxisomes. Occasionally, these peroxisomes show directed, long‐range movements with the linear protrusion leading (Figure 5C 28‐40 seconds; Video S10). These structures resemble the globular peroxisomes and protrusions induced by MIRO1 via microtubule‐dependent motor forces. Globular peroxisomes can become more static, either by docking to microtubules and/or tethering to other organelles such as the ER (66.2% ± 2.6% of peroxisomes in COS‐7 cells are associated with the ER^46^; Figure 5C 42‐54 seconds; Video S10). However, in contrast to the more static globular peroxisomes, the membrane protrusions show a more random, tentacle‐like movement, which does not seem to be directed by microtubules (Figure 5C 42‐54 seconds; Video S10). This type of movement likely allows the peroxisomes to efficiently explore the environment and to engage with other organelles while being attached. Peroxisomes can then detach again and continue to move in a directed manner. Occasionally, membrane division is observed (Figure 5C 135‐141 seconds; Video S10). These observations indicate that different, but cooperating mechanisms contribute to peroxisome dynamics and proliferation: PEX11β enables peroxisome membrane protrusion via its membrane deforming and scaffolding properties, and subsequently leads to division whereas MIRO1 on the other hand can elongate and divide peroxisomes by pulling forces via its interaction with microtubule‐dependent motors. Both mechanisms can function independently, as peroxisomes can elongate and divide in the absence of microtubules, but adaptors such as MIRO1 and associated motors provide directionality to peroxisome membrane expansion and peroxisome movement.

## 3 DISCUSSION

Our findings support a peroxisomal and mitochondrial localisation of MIRO1 and a role for MIRO1 in establishing peroxisome‐motor protein associations in mammalian cells. As MIRO1 can alter peroxisome distribution and motility, it is likely one of the yet unidentified adaptors for microtubule‐based peroxisome motility in mammalian cells. This assumption is further supported by recent findings showing that mitochondria and peroxisomes share many TA membrane proteins and their functions due to their close cooperation and co‐evolution in mammalian cells.^34^ During the submission of our work, Okumoto et al 55 revealed that distinct MIRO1 splice variants show different targeting to mitochondria and peroxisomes in HEK cells, with MIRO1‐variant 4 being more specific for peroxisomes. Peroxisomal MIRO1 also induced peroxisome accumulation and mediated long‐range movement of peroxisomes along microtubules further supporting a role for MIRO1 in peroxisomal motility. In contrast to our findings, MIRO1‐var1 localised primarily to mitochondria, which may be explained by the use of different cell lines or differences in expression levels of MIRO1.

We also show that peroxisome‐targeted MIRO1 can be used as a tool to exert pulling forces at peroxisomes, and that MIRO1‐mediated pulling forces have an impact on peroxisomal distribution, membrane dynamics and proliferation. These observations in combination with our mathematic model of peroxisome dynamics, shed light on the role of pulling forces in peroxisome formation by growth and division which have been controversial.^9^, ^17^ We show that MIRO1‐mediated motor forces along microtubules can elongate and divide peroxisomes. As elongation and division can still occur in the absence of microtubules, we suggest that independent, but cooperative mechanisms exist, and that motor forces support membrane dynamics by providing directionality. This is now in agreement with observations in yeast, where actin‐based, myosin‐driven pulling forces cause peroxisome elongation and separation in dynamin mutants.^56^, ^57^ Our approaches also contribute to the understanding of the versatility of peroxisome morphology in mammalian cells. In our model, we develop basic principles for peroxisome dynamics which govern peroxisome morphology. This helps us to understand why peroxisomes in division‐incompetent cells are highly elongated (due to continued lipid flow, eg, from the ER, in the absence of membrane fission), and why MIRO1‐mediated pulling forces can proliferate peroxisomes in fibroblasts (due to peroxisome‐ER tethers which prevent movement to the cell periphery). It also contributes to our understanding of peroxisome phenotypes in disease, eg, in PEX5 deficient cells from Zellweger patients. Here, metabolically compromised peroxisomes retain their plasticity and can elongate via MIRO1‐mediated pulling forces, but proliferation is reduced, likely due to altered membrane lipids.^51^

Despite their fundamental importance to cell physiology, the mechanisms that mediate and regulate peroxisomal membrane dynamics and abundance in humans are poorly understood. Our study aids in understanding these mechanisms which is not only important for comprehending fundamental physiological processes but also for understanding pathogenic processes in disease aetiology.

## 4 MATERIALS AND METHODS

### 4.1 Plasmids and antibodies

For cloning of peroxisome‐targeted MIRO1, the C‐terminal TMD and tail of Myc‐MIRO1 were exchanged by a PEX26/ALDP fragment previously shown to target proteins to the peroxisomal membrane.^39^ See Table S1 for details of plasmids used in this study, Table S2 for plasmids generated in this study and Table S3 for details of primers used. All constructs produced were confirmed by sequencing (Eurofins Genomics). Details on all antibodies used in this study can be found in Table S4.

### 4.2 Cell culture and transfection

COS‐7 cells (American Type Culture Collection (Rockville, MD) [ATCC] CRL‐1651), human control skin fibroblasts (C109), PEX5 and PEX14 deficient fibroblasts (provided by H. Waterham, AMC, University of Amsterdam, NL and M. Fransen, KU Leuven, BE) were cultured in DMEM, high glucose (4.5 g/L) supplemented with 10% FBS, 100 U/mL penicillin and 100 μg/mL streptomycin at 37°C with 5% CO_2_ and 95% humidity. WT and MIRO1 KO mouse embryonic fibroblasts (MEF) (provided by J. Shaw, University of Utah, USA) were cultured in the same media and supplemented with β‐mercaptoethanol at a final concentration of 50 μM. COS‐7 cells were transfected using TurboFect (Thermo Fisher Scientific). To analyse the effects of microtubule depolymerisation, cells were treated 24 hours after transfection with 10 μM nocodazole (10 mM stock in dimethyl sulphoxide [DMSO]) and incubated for 1 or 4 hours before being fixed. Control cells were incubated with the same volume of DMSO as that used to dissolve nocodazole (maximum 0.1% vol/vol). Fibroblasts were transfected by microporation using the Neon Transfection System (Thermo Fisher Scientific) following the manufacturer’s protocol. In short, cells (seeded 24 hours prior to transfection) were washed once with PBS and trypsinized using TrypLE Express. Trypsinized cells were resuspended in complete media without antibiotics and centrifuged for 3 minutes at 1000 rpm, and the pellet washed with PBS. The cells were once again centrifuged and carefully resuspended in 10 μL Buffer R. For each condition, 10^5^ cells were mixed with the DNA construct (1‐2 μg). Cells were microporated using a 10 μL Neon tip with the following settings: 1700 V, 20 ms, 1 pulse (human fibroblasts); 1350 V, 30 ms, 1 pulse (MEFs). Microporated cells were immediately seeded into plates with pre‐warmed complete medium without antibiotics and incubated at 37°C with 5% CO_2_ and 95% humidity. For live‐cell imaging, cells were co‐transfected with a fluorescent peroxisome marker (EGFP‐SKL for COS‐7 and EGFP‐ACBD5^TMD‐T^ for fibroblasts) at a 1:2 ratio with Myc‐MIRO1^Pex^ plasmids. As peroxisomal matrix import is defective in PEX5 and PEX14 patient fibroblasts, we used a fusion of EGFP and the TMD/tail region of ACBD5 (EGFP‐ACBD5^TMD‐T^) to label the peroxisomal membrane in control and patient cells.

### 4.3 Immunofluorescence and microscopy

Cells were processed for immunofluorescence 24 or 48 hours after transfection. Cells grown on glass coverslips were fixed for 20 minutes with 4% paraformaldehyde (PFA) in PBS (pH 7.4), permeabilized with 0.2% Triton X‐100 for 10 minutes and blocked with 1% BSA for 10 minutes. To visualise both peroxisomes and the microtubule network, cells were fixed for 10 minutes with 4% PFA followed by 5 minutes with ice‐cold methanol. Blocked cells were sequentially incubated with primary and secondary antibodies for 1 hour in a humid chamber (Table S4). Coverslips were washed with ddH_2_O to remove PBS and mounted with Mowiol medium on glass slides. All immunofluorescence steps were performed at room temperature and cells were washed 3 times with PBS between each individual step. Cell imaging was performed using an Olympus IX81 microscope equipped with an UPlanSApo 100×/1.40 oil objective (Olympus Optical). Digital images were taken with a CoolSNAP HQ2 CCD camera and adjusted for contrast and brightness using the Olympus Soft Imaging Viewer software (Olympus Soft Imaging Solutions GmbH) and MetaMorph 7 (Molecular Devices). Confocal images were obtained using a Zeiss LSM 510 META inverted microscope equipped with a Plan Apochromat 63×/1.4 NA (oil/dic) objective (Carl Zeiss), using the Argon 488 nm and He 543 nm laser lines. Digital images were adjusted for contrast and brightness using the Zeiss LSM Image Browser software (Carl Zeiss MicroImaging GmbH). Live‐cell imaging data was collected using an Olympus IX81 microscope equipped with a Yokogawa CSUX1 spinning disk head, CoolSNAP HQ2 CCD camera, 60×/1.35 oil objective. Digital images were taken and processed using VisiView software (Visitron Systems). For live‐cell imaging, cells were plated in 3.5 cm diameter glass bottom dishes (Cellvis and MatTek). Prior to image acquisition, a controlled temperature chamber was set‐up on the microscope stage at 37°C, as well as an objective warmer. During image acquisition, cells were kept at 37°C and in CO_2_‐independent medium (HEPES buffered). For COS‐7 cells, 500 stacks of 5 planes (0.5 μm thickness, 100 ms exposure) were taken in a continuous stream. For human fibroblasts, 250 stacks of 9 planes (0.5 μm thickness, 100 ms exposure) were taken in a continuous stream. All conditions and laser intensities were kept between experiments. For each condition analysed, a representative cell was selected and the acquired images were converted into a video at 10× the original speed.

### 4.4 Peroxisome motility, number and length measurements

Peroxisomes were automatically detected and tracked using a customised in‐house algorithm.^41^, ^46^ Briefly, each image was filtered using a scale‐space Laplace of Gaussian filtering approach^58^, ^59^ over scales corresponding to the size range of peroxisomes. After filtering, a threshold was determined using the median absolute deviation as a robust estimator of the background level,^60^ and applied to the filter response to determine peroxisome positions. Once detected, peroxisomes were tracked using a global optimization subroutine (using a modified version of the Jonker‐Volgenant algorithm).^61^ Tracking results were manually verified for accuracy. For trajectory plots, 100 trajectories were retrieved for each condition by randomly selecting approximately 4 trajectories, of length at least 20 time‐frames, from each data set. Next, the trajectories were re‐centred such that each trajectory started at (0,0), and subsequently smoothed applying a simple moving‐average algorithm using a Hann window. The first 20 time‐frames for these trajectories were then plotted starting at a centre. For cumulative distribution function (CDF) plots, basic instantaneous trajectory speed profiles were estimated by calculating the distance moved between each time‐point in the trajectory. These speeds were then pooled and converted into an ECDF. By pooling the speeds for all data sets for a given condition a single ECDF for each condition was generated. Trajectories for the tracked peroxisomes were analysed by splitting their instantaneous speeds into 2 groups, using a cut‐off for linear motion speed of 0.24 μm/s.^42^ The relative populations of the 2 groups of peroxisome speeds were used as an indication of the amount of linear motion for each data set, and compared against all trajectories to obtain a percentage of microtubule‐dependent motility per cell. The number of peroxisomes per cell was obtained from the motility analysis output, and determined by the detected peroxisome from the first frame of each analysed cell. Peroxisome protrusion lengths were obtained from live‐cell imaging data and manually measured using MetaMorph 7. Each observed protrusion was measured at the longest point of extension. Kymographs were generated using ImageJ (developed at the National Institutes of Health).

### 4.5 Immunoprecipitation

For immunoprecipitation experiments Myc‐MIRO1 WT and HA‐PEX19 were expressed in COS‐7 cells. After 48 hours cells were washed in PBS and incubated with 1 mM DSP followed by quenching with 100 mM Tris pH 7.4. After crosslinking cells were lysed in ice‐cold lysis buffer (25 mM Tris‐HCl pH 7.5, 150 mM NaCl, 0.5% Triton X‐100, 1 mM phenylmethylsulfonyl fluoride (PMSF) and protease inhibitor cocktail). Undissolved material was pelleted by centrifugation at 15000*g.* The supernatant was mixed with Myc‐antibody coupled agarose beads and incubated for 2 hours at 4°C. Beads were subsequently washed extensively with lysis buffer by quick centrifugations at 12000*g* and by incubating in a rotating shaker for 15 minutes at 4°C. Bound proteins were eluted with 50 mM NaOH and the eluted protein was denatured in Laemmli buffer for 10 minutes at 95°C. Immunoprecipitates and total lysates were analysed by Western immunoblotting.

### 4.6 Mathematical modelling

Each peroxisome was described by its body radius *r* and elongation length *L.* Simulations were started with 250 peroxisomes, each with a random initial radius and no elongation. After each time step (*∆t* = 1 second), we implemented 3 processes. First, lipid flow from the ER into the body: the body surface area was increased by *α∆t* with probability *e* ^*−γA*^, where *A* is the total area of all peroxisomes. Second, if the body radius was above *r*_min_, the elongation was increased by length *v∆t*, with the extra elongation area taken from the body. Third, when the elongation length was longer than *L*_min_, peroxisomes underwent division with probability *βL∆t.* In addition, during each time step, each peroxisome had probability *∆t/τ* of being removed by turnover. Simulations were carried out in C++ and MATLAB. See Supporting information for full details. The MATLAB code can be made available upon request.

### 4.7 Statistical analyses

For quantitative analysis of the effect of MIRO1 expression on peroxisome distribution, motility and number, at least 3 independent experiments were carried out. Statistical analyses were performed using Microsoft Excel and GraphPad Prism 5 software. Data are presented as means ± SEM. Two‐tailed unpaired *t* tests and one‐way ANOVA with post hoc Tukey tests were used to determine statistical differences against control values. * *P* < .05, ** *P* < .01, *** *P* < .001.

## Supporting information


**Supporting Information**

**Figure S1.** A, COS‐7 cells transfected with Myc‐MIRO1 wild type and mutants were fixed and stained against Myc and PEX14. Expressed Myc‐MIRO1 localises to peroxisomes and mitochondria, and alters their distribution. All of the expressed mutants show peroxisomal (and mitochondrial) localisation, except for Myc‐MIRO1^ΔTM^, which is cytosolic. Bars, 20 μm (overview), 5 μm (magnification). B, Quantitative analysis of peroxisome distribution in controls and cells expressing different Myc‐MIRO1 plasmids. Cells with peroxisomal accumulations in the periphery or scattered were counted. Values represent mean ± SEM of 3 independent experiments (100 replicates per experiment per condition; ** *P* < .01; *** *P* < .001; one‐way ANOVA with post hoc Tukey test vs control cells).

**Figure S2.** A, COS‐7 cells were transfected with Myc‐MIRO1^Pex^ and, after 24 hours, treated with 10 μM nocodazole or DMSO (control) for 4 hours. Fixed cells were stained against Myc and TUBULIN. Cells expressing Myc‐MIRO1^Pex^ no longer showed peroxisome aggregates at the cell periphery after treatment with nocodazole. Note that microtubule depolymerisation can lead to peroxisomal aggregates in the cytoplasm. B, Immunoblot of cell lysates from MIRO1 KO and control mouse embryonic fibroblasts (MEFs) stained against MIRO1 and γ‐TUBULIN. C, Control and MIRO KO MEFs were transfected with EGFP‐SKL and fixed after 24 hours. Bars, 20 μm. D, Control and MIRO1 KO MEFs were transfected with EGFP‐SKL. For each cell analysed, 250 stacks of 9 planes were obtained over time. Percentage of fast moving peroxisomes per cell in control (5.54 ± 0.95) and MIRO1 KO cells (5.28 ± 0.93). Values represent mean ± SEM of 8 to 12 cells in 1 experiment. E‐H, CDF plot. Instantaneous trajectory speed profiles were estimated by calculating the distance moved between each time point in the trajectory. These speeds were pooled and converted to an ECDF. By pooling speeds for all data sets for a given condition, a single ECDF was generated for each. A threshold of 0.24 μm/s was defined for microtubule‐dependent motility. E, Control and MIRO1 KO MEFs. F, C109 fibroblasts. G, dPEX5 fibroblasts. H, dPEX14 fibroblasts.

**Table S1.** Plasmids used in this study.
**Table S2.** Plasmids generated in this study.
**Table S3.** Primers used in this study.
**Table S4.** Primary and secondary antibodies used in this study.

**Video S1.** Myc‐MIRO1^V13‐Pex^ expression increases peroxisome movement (control). COS‐7 cell transfected with EGFP‐SKL; 250 frames, 10× speed.

**Video S2.** Myc‐MIRO1^V13‐Pex^ expression increases peroxisome movement (Myc‐MIRO1^V13‐Pex^). COS‐7 cell transfected with EGFP‐SKL and Myc‐MIRO1^V13‐Pex^; 250 frames, 10× speed.

**Video S3**. Myc‐MIRO1^Pex^ expression increases peroxisome number and motility in human fibroblasts (control). C109 cell transfected with EGFP‐ACBD5^TMD‐T^; 125 frames, 10× speed.

**Video S4**. Myc‐MIRO1^Pex^ expression increases peroxisome number and motility in human fibroblasts (Myc‐MIRO1^Pex^). C109 cell transfected with EGFP‐ACBD5^TMD‐T^ and Myc‐MIRO1^Pex^; 125 frames, 10× speed.

**Video S5.** Myc‐MIRO1Pex expression increases peroxisome number and motility in human fibroblasts (control). dPEX5 cell transfected with EGFP‐ACBD5TMD‐T; 125 frames, 10× speed.

**Video S6**. Myc‐MIRO1^Pex^ expression increases peroxisome number and motility in human fibroblasts (Myc‐MIRO1^Pex^). dPEX5 cell transfected with EGFP‐ACBD5^TMD‐T^ and Myc‐MIRO1^Pex^; 250 frames, 10× speed.

**Video S7** Myc‐MIRO1^Pex^ expression increases peroxisome number and motility in human fibroblasts (Control). dPEX14 cell transfected with EGFP‐ACBD5^TMD‐T^; 125 frames, 10× speed.

**Video S8** Myc‐MIRO1^Pex^ expression increases peroxisome number and motility in human fibroblasts (Myc‐MIRO1^Pex^). dPEX14 cells were transfected with EGFP‐ACBD5^TMD‐T^ and Myc‐MIRO1^Pex^; 125 frames, 10× speed.

**Video S9** Myc‐MIRO1Pex expression induces the formation of peroxisome elongations in dPEX5 patient fibroblasts (cut‐out of Video S6). dPEX5 cell transfected with EGFP‐ACBD5TMD‐T and Myc‐MIRO1Pex; 160 frames, 10× speed.

**Video S10** PEX11β‐EGFP induces peroxisome elongation and proliferation in COS‐7 cells. COS‐7 cell transfected with PEX11β‐EGFP. Two hundred stacks of 9 planes (0.5 μm thickness, 100 ms exposure) were taken in a continuous stream; 200 frames, 10× speed.
