# Supplementary material for: A role for Mitochondrial Rho GTPase 1 (MIRO1) in motility and membrane dynamics of peroxisomes
Source: Traffic. 2018 Feb 20;19(3):229–42. doi: 10.1111/tra.12549 (PMC5888202; doi:10.1111/tra.12549)
Supplement: Supplementary file 1 — Supporting Information [file TRA-19-229-s001.pdf]

Supporting Text for *A role for MIRO1 in motility and membrane dynamics of peroxisomes*

1 Details of mathematical model

Our model describes the shape and number of peroxisomes within a cell. It is a stochastic, population-based model that considers the morphology of a group of peroxisomes, each of which can grow, divide and turnover. Since our aim is to understand the basic biophysical mechanisms involved in peroxisome growth and proliferation, we focus on the peroxisome shape and ignore both their spatial distribution and interactions between each other. Further, we only include a few key processes that affect peroxisome size and number (such as lipid flow and division). This is a deliberate decision that aims to tease out the main factors responsible for peroxisome morphology.

Although we acknowledge that real peroxisome dynamics is likely to be considerably more complex than this, we believe that simpler models with fewer parameters are more valuable and predictive, especially as this is, as far as we know, the first model describing peroxisome shape. The intention is that later models will be able to build on the work described here by including other relevant aspects (such as spatial distribution and interaction).

1.1 Description of model

We describe each peroxisome as consisting of a spherical body of radius r, with the centre at some position $\vec{x}$. In addition, each peroxisome has an optional elongation of length L, emanating at some fixed angle from the body. This elongation is assumed to be straight and to have constant diameter w, so that it can be modelled as a cylinder with a hemispherical cap. Although it is clear that not all real peroxisomes can be described by such a body-elongation arrangement, and although real elongations often bend and have variable width, this idealised representation is sufficient for our present purposes.

We consider three processes by which peroxisomes can change shape: membrane lipid flow, elongation growth, and division. Firstly, peroxisomes are assumed to grow due to membrane lipids flowing from the ER via membrane contacts [1]. Since there is not unlimited lipid, this process is not always successful. To model this we assume that, at any given time, there is some probability $p = e^{-\gamma A}$ that lipid flow occurs, where A is the total surface area of all peroxisomes and γ a constant that we call the lipid flow constant. This means that, in our model, lipid flow is always possible but is less likely

as the total peroxisomal content increases. This assumption that lipid flow depends only on total peroxisome area is simplistic but sufficient for a first model. A more complex model could take account of the spatial distribution of peroxisomes relative to the ER and to *de novo* lipid production. Although lipid flow is unlikely to be constant in practice, we assume for simplicity that there is some fixed rate α at which membrane area tries to flow into the peroxisome. Since peroxisomes without elongations still grow, it is likely that the link to the ER is located somewhere within the body and so we assume that lipid flow only increases the radius of the body without changing the elongation length. Thus, in some time t, the effect of successful lipid flow is to increase r to $\sqrt{r^2 + \alpha t/(4\pi)}$ whilst leaving L unchanged.

Secondly, we consider growth of the elongation. New peroxisomes initially have no elongation ($L = 0$) and must wait until the body radius is above some minimum size r_{min} before the elongation forms. However, once formed, whenever the body radius is above r_{min}, the elongation then extends at fixed speed v. At the moment a new elongation appears, it consists only of a hemispherical cap of radius $w/2$, which replaces a spherical cap on the body of base radius $w/2$. The membrane area to create this cap is assumed to come from the body, so that r decreases to $\sqrt{r^2 - w^2/8 + \frac{1}{2}r^2(1 - \sqrt{1 - w^2/4r^2})}$ at the moment of formation. Here, the second term corresponds to the surface area of the hemispherical cap and the third term to the surface area of the removed spherical cap ($A_{\text{spherical cap}} = 2\pi r h$, where h is the cap height). The exact mechanism of extension, such as Pex11β oligomerisation or MIRO1-driven motion along microtubules, need not be explicitly specified: from a modelling perspective these mechanisms only differ in the value of v. A more realistic model could consider a variable speed of extension, but this is unlikely to play an important role here. The membrane area required in order to extend the elongation is assumed to come from the body so that the overall membrane area is conserved during the extension process. This means that, after extending for time t, the elongation increases in length to $L + vt$ with a compensating reduction in body size from r to $\sqrt{r^2 - wvt/4}$. The second term originates from the new surface area (πwvt) of the cylindrical elongation.

Thirdly, we include peroxisome division, which occurs by the elongation splitting off to form new peroxisomes. We assume that this can only occur if the elongation length L is above some minimum value L_{min}. We further assume that peroxisomes with longer elongations are more likely to divide, which we implement via a fixed rate of division per unit length, β. We interpret this in a probabilistic manner so that in a small time dt the probability of division is $\beta L dt$. Upon division, the elongation is separated from

the body, split into regularly-sized compartments of length λ, with each becoming a new "daughter" peroxisome. The body of the original peroxisome remains as the "mother" peroxisome. The small cylinder that forms each "daughter" becomes the body of the new peroxisome (with membrane area conserved). After division the "mother" and all "daughter" peroxisomes are without elongations ($L = 0$) and must wait until $r \geq r_{\min}$ before new elongations can form.

In addition, to avoid the number of peroxisomes increasing without limit, we include peroxisome turnover by introducing a mean lifetime τ for each peroxisome. This is implemented as a probability $p = dt/\tau$ that any given peroxisome degrades in a time dt. Finally, the position $\vec{x}$ and elongation angle of each peroxisome undergo diffusion. This is not a crucial part of the current model and is included mainly to improve visualisation of the results. Here we are chiefly interested in the shape of peroxisomes rather than their spatial position. We leave the important issue of spatial distribution for future models.

1.2 Numerical simulation

Each simulation starts with $n = 250$ peroxisomes, with each having a random initial radius and position, and no elongation ($L = 0$). The initial radius is chosen from a truncated normal distribution with mean 70nm, standard deviation 30nm, minimum 40nm and maximum 100nm. At each time step ($\Delta t = 1$s) we implement the three processes described above: (i) r is increased due to membrane lipid flow with probability $e^{-\gamma A}$, (ii) L is increased and r decreased due to elongation growth (for those peroxisomes that are sufficiently large), and (iii) there is a random chance of division (for those peroxisomes with sufficiently long elongations). Further, each peroxisome has probability $\Delta t/\tau$ of undergoing turnover during each time step. The simulation is run for at most 10 days and is stopped once an approximate steady state has been reached (characterised by sufficiently small variations in n, $\langle r \rangle$ and $\langle L \rangle$ over the last six hours).

1.3 Parameter values

At any given time we expect a population of peroxisomes of various sizes and shapes. In particular, at steady state, let n be the number of peroxisomes, f the fraction of peroxisomes that have elongations, $\langle r \rangle$ the average body radius, and $\langle L \rangle$ the average elongation length of those peroxisomes with elongations (*i.e.* of those peroxisomes with $L > 0$). From EM and IMF images of wild-type cells, we estimated that $n \sim 250$, $f \sim 0.1$, $\langle r \rangle \sim 80$nm

and $\langle L \rangle \sim 40$nm. Although these values are likely to vary between different cell types and organisms, they are sufficient for our purpose here, an initial model that sheds light on the biophysical processes involved and recapitulates various mutant phenotypes.

The following sections describe how the wild-type model parameters were estimated. Note that, although it may be possible to find parameters that better fit the data, the fact that (i) these parameters lead to a good match with the wild type, (ii) the model can incorporate overexpression of MIRO1, and (iii) changing only one or two parameters can capture the MFF and PEX5 deficient phenotypes, suggests that this parameter set is sufficient for this first generation model.

1.3.1 The elongation diameter w and compartment length λ

Firstly, from EM images, we measured the average elongation diameter to be about $w = 80$nm [1, 2]. Then, since the smallest new peroxisomes have initial radius $\sqrt{w\lambda/4}$ and since we rarely see peroxisomes smaller than 40nm, this suggests $\lambda \sim 80$nm.

1.3.2 The minimum elongation length for division $L_{\min}$

Next, assuming that most peroxisomes divide very soon after L reaches $L_{\min}$ (which is appropriate for the wild type), the average elongation length $\langle L \rangle \approx \frac{1}{2} L_{\min}$. (This is only approximately true since turnover means that peroxisomes with longer elongations are rarer than those with shorter elongations.) This motivates setting $L_{\min} = 80$nm. Coincidentally, this means that $L_{\min} = \lambda$, which makes sense since then new peroxisomes smaller than 40nm in radius cannot be formed.

1.3.3 The minimum radius for elongation extension $r_{\min}$

By assuming that, for elongating peroxisomes, all available lipid flowing into the body is always used only for elongation growth (*i.e.* $\pi w v > \alpha$), the average body radius can be estimated as $\langle r \rangle \sim (1 - f)(\sqrt{w\lambda/4} + r_{\min})/2 + f r_{\min} = \frac{1}{4}(1 - f)\sqrt{w\lambda} + \frac{1}{2}(1 + f) r_{\min}$. Again, this is only an approximation since the distribution of peroxisome body sizes is not uniform. Using an $\langle r \rangle$ of 80nm then leads to $r_{\min} \sim 110$nm.

1.3.4 The peroxisome mean lifetime τ

Next, to estimate τ, consider overall lipid flow. In steady state, during some time Δt, the total lipid entering the system must exactly balance the lipid

leaving the system. Since the total lipid area entering (due to lipid flow into the body) is approximately $n \cdot \exp(-\gamma n\langle A\rangle) \cdot \alpha\Delta t$, and since the total leaving (due to turnover) is approximately $n \cdot \frac{\Delta t}{\tau} \cdot \langle A\rangle$, we find that $\langle A\rangle = \alpha\tau \exp(-\gamma n\langle A\rangle)$. Then the time between a new peroxisome forming and first starting to elongate, $\pi(4r_{\min}^2 - w\lambda)/(\alpha \exp(-\gamma n\langle A\rangle))$, can be rewritten as $\pi\tau(4r_{\min}^2 - w\lambda)/\langle A\rangle$. Taking a value of 3 days for this, and estimating $\langle A\rangle$ as $4\pi\langle r\rangle^2 + f\pi w\langle L\rangle \approx 10^5\text{nm}^2$, we conclude that $\tau \sim 1.5 \times 10^5$s. This corresponds to a mean peroxisome lifetime of just under 2 days, which agrees well with previously measured values [3].

1.3.5 The division rate β

Given that, at least in the wild type, peroxisomes divide before the elongation length is much greater than $L_{\min}$, a peroxisome that reaches $L_{\min}$ will wait on average about $1/(\beta L_{\min})$ before division. The lack of significant peroxisome elongations in wild-type cells suggests this time is quite short. Taking a value of 10 mins for this, we estimate that $\beta = 2 \times 10^{-5}$/nm/s.

1.3.6 The lipid flow rate α and lipid flow constant γ

By considering only steady-state values, such as n, f, $\langle r\rangle$ and $\langle L\rangle$, it is not possible to uniquely fit α and γ. This is because these parameters effectively only ever appear together in the combination $\alpha \exp(-\gamma n\langle A\rangle)$, which is the maximum possible lipid flow rate multiplied by the probability of lipid flow actually occurring, and can be thought of as the "effective lipid flow rate". However, it is possible to fit one of these parameters by considering the transient period before steady state. With all other parameters fixed, the time to reach steady state varies inversely with α: smaller/larger values of α take longer/shorter to reach steady state. With the above parameters, our simulations show that choosing $\alpha = 75\text{nm}^2/\text{s}$ means that steady state is reached in a few tens of hours. Once α is fixed, the lipid flow constant γ follows from the overall-lipid-flow equation derived in §1.3.4, which gives $\gamma = \ln(\alpha\tau/\langle A\rangle)/(n\langle A\rangle) \sim 2.4 \times 10^{-7}\text{nm}^{-2}$.

1.3.7 The elongation extension speed v

Finally, we choose the elongation extension speed v so that elongation growth normally uses all the lipid flowing into the body. At steady state, this means that $\pi wv > \alpha \exp(-\gamma n\langle A\rangle)$. In fact, our simulations suggest that a value of $v \sim 0.3$nm/s gives a good match to the experimental data for the wild-type and the MFF and PEX5 deficient cases.

1.3.8 Summary

The following table lists all model parameters and their wild-type values.

Parameter	Wild-type value	Section
Lipid flow rate, α	$75\,\text{nm}^2/\text{s}$	§1.3.6
Elongation extension speed, v	$0.3\,\text{nm/s}$	§1.3.7
Division rate, β	$2 \times 10^{-5}/\text{nm/s}$	§1.3.5
Peroxisome mean lifetime, τ	$1.5 \times 10^5\,\text{s}$	§1.3.4
Lipid flow constant, γ	$2.4 \times 10^{-7}\,\text{nm}^{-2}$	§1.3.6
Elongation diameter, w	$80\,\text{nm}$	§1.3.1
Elongation comp. length, λ (sets new PO size)	$80\,\text{nm}$	§1.3.1
Minimum r for elongation extension, r_{min}	$110\,\text{nm}$	§1.3.3
Minimum L for division, L_{min}	$80\,\text{nm}$	§1.3.2

References

[1] Costello JL, Castro IG, Hacker C, Schrader TA, Metz J, Zeuschner D, Azadi AS, Godinho LF, Costina V, Findeisen P, Manner A, Islinger M, Schrader M (2017) ACBD5 and VAPB mediate membrane associations between peroxisomes and the ER. *J Cell Biol* 216(2):331–342.

[2] Delille HB, Agricola S, Guimaraes S, Borta H, Luers GH, Fransen M, Schrader M (2010) Pex11pβ-mediated growth and division of mammalian peroxisomes follows a maturation pathway. *J Cell Sci* 123(16):2750–62.

[3] Huybrechts SJ, Van Veldhoven PP, Brees C, Mannaerts GP, Los GV, Fransen M (2009) Peroxisome dynamics in cultured mammalian cells. *Traffic* 10(11):1722–33.
