# Supplementary figures and images for: A role for Mitochondrial Rho GTPase 1 (MIRO1) in motility and membrane dynamics of peroxisomes
Source: Traffic. 2018 Feb 20;19(3):229–42. doi: 10.1111/tra.12549 (PMC5888202; doi:10.1111/tra.12549)

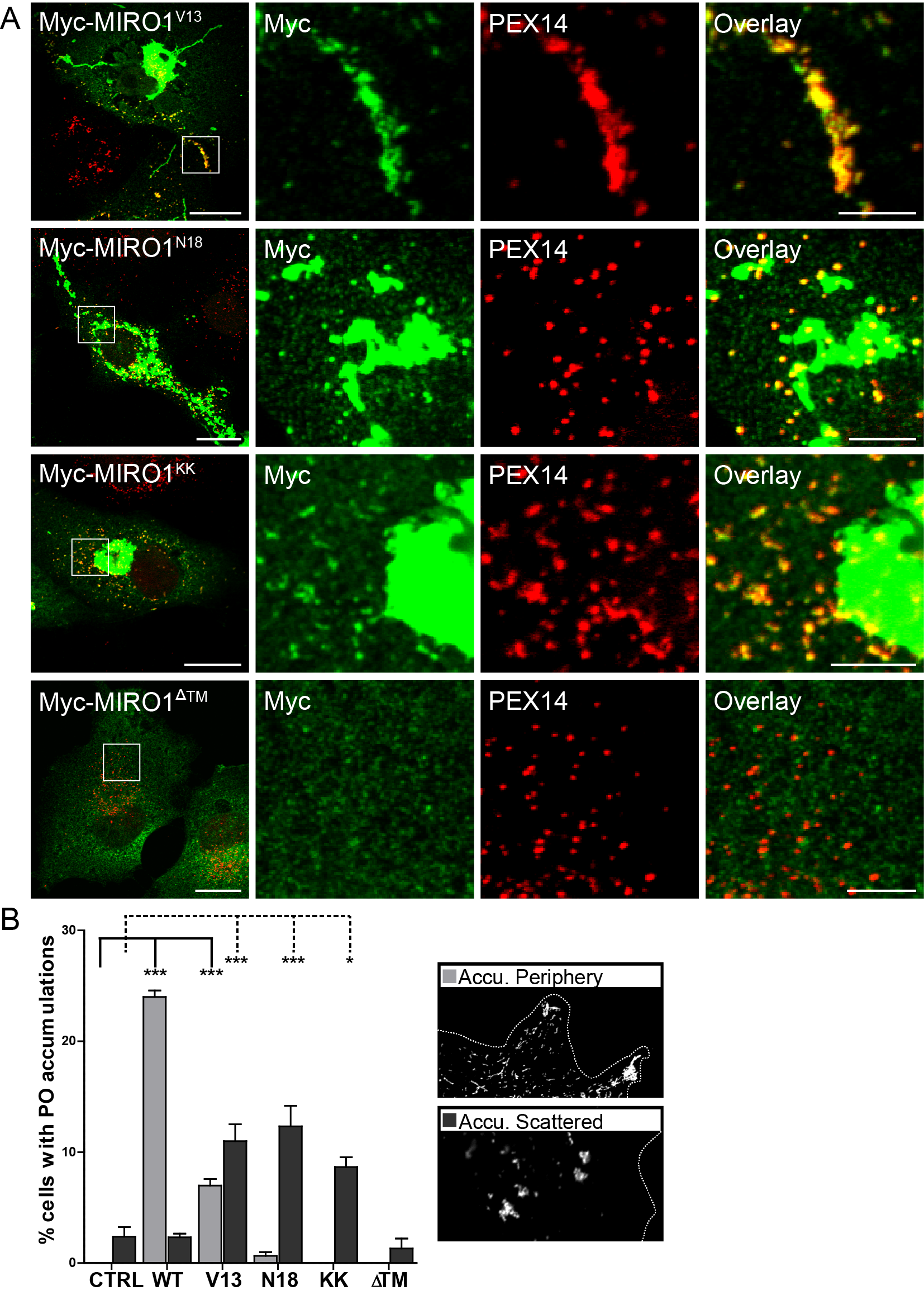

Supplement: Supplementary file 2 — Figure S1. A, COS‐7 cells transfected with Myc‐MIRO1 wild type and mutants were fixed and stained against Myc and PEX14. Expressed Myc‐MIRO1 localises to peroxisomes and mitochondria, and alters their distribution. All of the expressed mutants show peroxisomal (and mitochondrial) localisation, except for Myc‐MIRO1ΔTM, which is cytosolic. Bars, 20 μm (overview), 5 μm (magnification). B, Quantitative analysis of peroxisome distribution in controls and cells expressing different Myc‐MIRO1 plasmids. Cells with peroxisomal accumulations in the periphery or scattered were counted. Values represent mean ± SEM of 3 independent experiments (100 replicates per experiment per condition; ** P < .01; *** P < .001; one‐way ANOVA with post hoc Tukey test vs control cells). [file TRA-19-229-s002.tif]

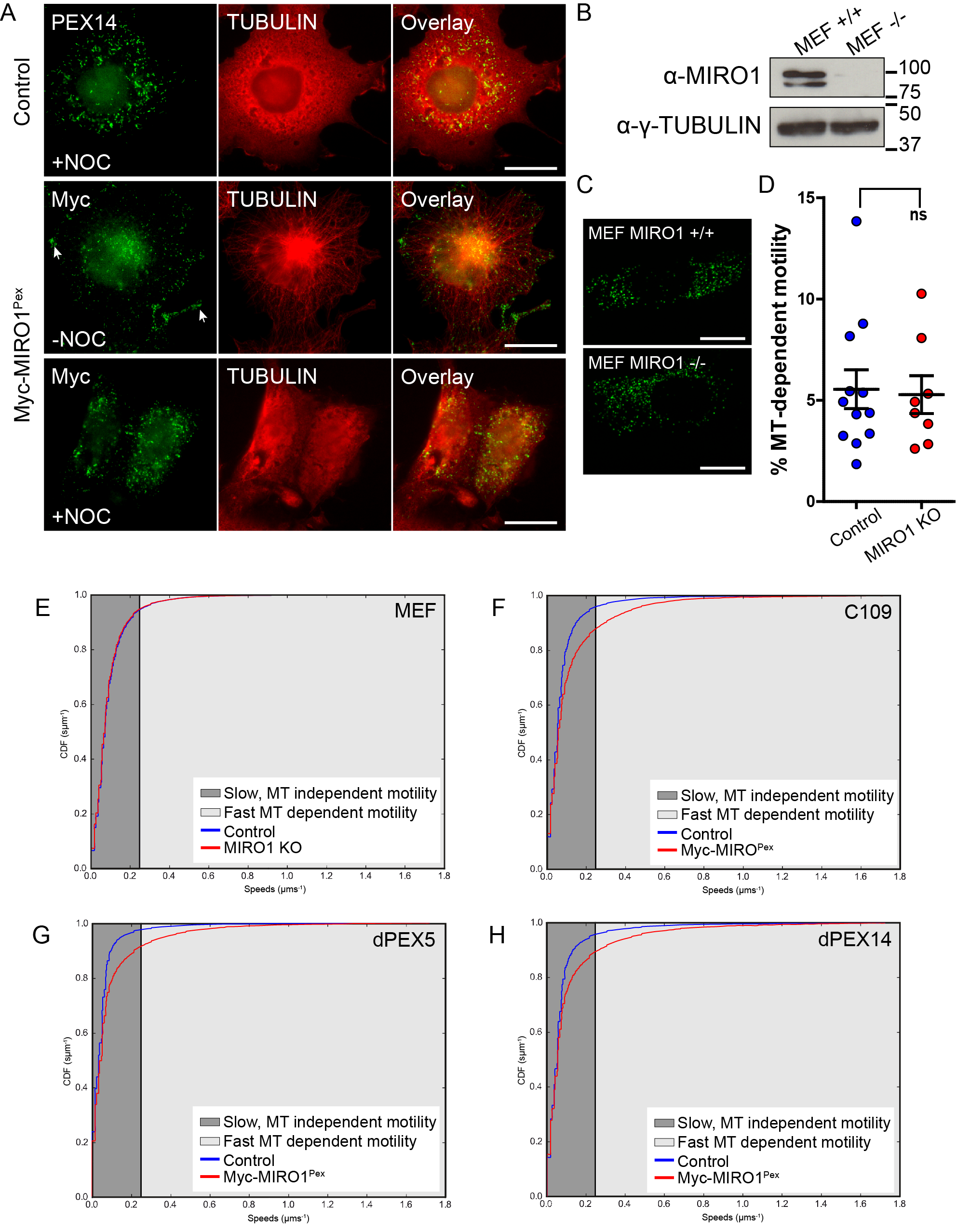

Supplement: Supplementary file 3 — Figure S2. A, COS‐7 cells were transfected with Myc‐MIRO1Pex and, after 24 hours, treated with 10 μM nocodazole or DMSO (control) for 4 hours. Fixed cells were stained against Myc and TUBULIN. Cells expressing Myc‐MIRO1Pex no longer showed peroxisome aggregates at the cell periphery after treatment with nocodazole. Note that microtubule depolymerisation can lead to peroxisomal aggregates in the cytoplasm. B, Immunoblot of cell lysates from MIRO1 KO and control mouse embryonic fibroblasts (MEFs) stained against MIRO1 and γ‐TUBULIN. C, Control and MIRO KO MEFs were transfected with EGFP‐SKL and fixed after 24 hours. Bars, 20 μm. D, Control and MIRO1 KO MEFs were transfected with EGFP‐SKL. For each cell analysed, 250 stacks of 9 planes were obtained over time. Percentage of fast moving peroxisomes per cell in control (5.54 ± 0.95) and MIRO1 KO cells (5.28 ± 0.93). Values represent mean ± SEM of 8 to 12 cells in 1 experiment. E‐H, CDF plot. Instantaneous trajectory speed profiles were estimated by calculating the distance moved between each time point in the trajectory. These speeds were pooled and converted to an ECDF. By pooling speeds for all data sets for a given condition, a single ECDF was generated for each. A threshold of 0.24 μm/s was defined for microtubule‐dependent motility. E, Control and MIRO1 KO MEFs. F, C109 fibroblasts. G, dPEX5 fibroblasts. H, dPEX14 fibroblasts. [file TRA-19-229-s003.tif]
