# Supplementary material for: A role for Mitochondrial Rho GTPase 1 (MIRO1) in motility and membrane dynamics of peroxisomes
Source: Traffic. 2018 Feb 20;19(3):229–42. doi: 10.1111/tra.12549 (PMC5888202; doi:10.1111/tra.12549)
Supplement: Supplementary file 4 — Table S1. Plasmids used in this study. Table S2. Plasmids generated in this study. Table S3. Primers used in this study. Table S4. Primary and secondary antibodies used in this study. [file TRA-19-229-s004.pdf]

Table S1. **Plasmids used in this study**

Plasmid	Source
EGFP-ACBD5^{TMD-T} WT	[34]
EGFP-SKL	[30]
HA-Pex19	[31]
Myc-MIRO1-WT, V13, N18, KK, ΔTM	P. Aspenström, Karolinska Institute, Sweden
pAH26	R. Erdmann, Univ. Bochum, Germany
PEX11β-EGFP	G. Dodt, Univ. of Tuebingen, Germany

Table S2. **Plasmids generated in this study**

Name	Template	Primers	Enzymes	Vector
Myc-MIRO1-Pex	Myc-Miro1	Myc-Miro1_AgeI_F<br>Miro1_dTM_R	AgeI<br>BglII	pAH26

Table S3. **Primers used in this study**

Name	Sequence (5’ to 3’)
Myc-Miro1_AgeI_F	GGAACCGGTCACCATGGAGCAGAAGCTGATC
Miro1-dTM_R	GGAAGATCTAAACGTGGAGCTCTTGGGGTC
Miro1seqmid1	CGCACAGAAAGCTGTTCTTCATCC
Miro1seqmid2	GACTGAGCAAGAGTCTCAAG

Table S4. **Primary and secondary antibodies used in this study**

Antibodies	Type	Dilution		Source
		IMF	WB	
HA	mc ms	-	1:1000	BioLegend
MIRO1 (PSI-8027)	pc rb	1:100	1:1000	ProSci
Myc (Ab9106)	mc rb	1:200	1:1000	Abcam
Myc 9E10	mc ms	1:200	1:1000	Santa Cruz Biotechonology
PEX14	pc rb	1:1400	1:4000	D.Crane, Griffith University, Australia
PMP70	mc ms	1:500	1:5000	Sigma-Aldrich
TOM20 (612278)	mc ms	1:200	-	BD Transduction Laboratories
γ-TUBULIN	mc ms	1:100	-	Sigma-Aldrich
AlexaFluor 488 IgG	dk anti-rb	1:500	-	ThermoFisher Scientific
AlexaFluor 488 IgG	dk anti-ms	1:500	-	ThermoFisher Scientific
AlexaFluor 594 IgG	dk anti-rb	1:1000	-	ThermoFisher Scientific
AlexaFluor 594 IgG	dk anti-ms	1:1000	-	ThermoFisher Scientific
HRP IgG	gt anti-ms	-	1:5000	Bio-Rad
HRP IgG	gt anti-rb	-	1:5000	Bio-Rad

Abbreviations: IMF, immunofluorescence; WB, western blot; mc, monoclonal; pc, polyclonal; ms, mouse; rb, rabbit; gt, goat; dk, donkey; HRP, horseradish peroxidase.
